# Analysis of clinical characteristics and prognosis of lung cancer patients with CPFE or COPD: a retrospective study

**DOI:** 10.1186/s12890-024-03088-5

**Published:** 2024-06-08

**Authors:** Yuying Wei, Liuqing Yang, Qing Wang

**Affiliations:** Department of Respiratory Medicine, The First Affiliated Hospital, College of Medicine, Zhejiang University, No.79, Qingchun Road, Hangzhou, Zhejiang China

**Keywords:** Lung cancer (LC), Combined pulmonary fibrosis and emphysema (CPFE), Chronic obstructive pulmonary disease (COPD), Progression-free survival (PFS)

## Abstract

**Background:**

Lung cancer (LC) commonly occurs in patients with combined pulmonary fibrosis and emphysema (CPFE) and chronic obstructive pulmonary disease (COPD), but comparative research is limited. This study examines clinical characteristics, treatments, and prognosis in LC patients with CPFE or COPD.

**Methods:**

The retrospective study involved 75 lung cancer patients with CPFE and 182 with COPD. It analyzed clinical features, tumor pathology, pulmonary function, laboratory parameters, and treatment responses.

**Results:**

Notable differences were found between the CPFE + LC and COPD + LC groups. Both groups were mostly elderly, male smokers. The CPFE + LC group had higher BMI and more adenocarcinoma and squamous cell carcinoma, while COPD + LC had predominantly squamous cell carcinoma. CPFE + LC tumors were mostly in the lower lobes; COPD + LC’s were in the upper lobes. The CPFE + LC group showed higher tumor metastasis rates, more paraseptal emphysema, and elevated levels of TG, CEA, NSE, and Killer T Cells. In advanced stages (IIIB-IV), the CPFE + LC group receiving first-line treatment had shorter median progression-free survival (PFS) and a higher risk of progression or death than the COPD + LC group, regardless of whether it was non-small cell lung cancer (NSCLC) or small cell lung cancer (SCLC). No significant PFS difference was found within CPFE + LC between chemotherapy and immunotherapy, nor in immune-related adverse events between groups, with interstitial pneumonia being common.

**Conclusion:**

This study emphasizes distinct lung cancer characteristics in CPFE or COPD patients, highlighting the need for tailored diagnostic and treatment approaches. It advocates for further research to improve care for this high-risk group.

## Introduction

Lung cancer (LC) is a global healthcare concern, representing the most prevalent form of cancer worldwide, accounting for 11.6% of all cancer cases and standing as the leading cause of cancer-related fatalities. In 2018, over 1.7 million lives were claimed by lung cancer [1, 2].

Chronic obstructive pulmonary disease (COPD) is associated with a high disease burden, and according to predictions by the World Health Organization, it will become the third leading cause of death by 2030 [3]. Both lung cancer and COPD are highly associated with smoking, and COPD is an independent risk factor for the development of lung cancer [4]. Reports indicate that COPD affects a significant proportion of lung cancer patients worldwide, ranging from 45 to 63% [5].

Combined pulmonary fibrosis and emphysema (CPFE) is a unique clinical entity [6], with a prevalence of 26–54% among patients with idiopathic pulmonary fibrosis (IPF) [7–9]. According to an Official ATS/ERS/JRS/ALAT Clinical Practice Guideline, approximately 2–52% of CPFE patients eventually develop lung cancer [7]. High-resolution computed tomography (HRCT) shows emphysema in the upper lobes of the lung along with fibrosis in the lower lobes [6]. CPFE often presents with impaired gas exchange, and is prone to complications such as pulmonary hypertension and lung cancer [6, 10, 11].

Both COPD and CPFE represent chronic lung diseases that are frequently encountered among elderly male smokers [7]. They share radiological evidence of pulmonary emphysema, and the incidence of concurrent lung cancer is significantly elevated in both conditions. However, patients with CPFE or COPD demonstrate notable differences in terms of pathology, physiology, clinical presentation, radiology, and prognosis [12]. Currently, research comparing lung cancer patients with CPFE or COPD remains limited, underscoring the need for more extensive exploration.

This study aims to delve into the clinical characteristics and treatment outcomes of lung cancer patients with CPFE or COPD using a retrospective approach based on real-world data. This research not only helps in comprehensively understanding these two complex diseases but also holds significant importance in developing precision medicine strategies for these high-risk groups.

## Methods

### Study design and patient selection

This retrospective study included 75 lung cancer patients with CPFE (CPFE + LC group) and 182 lung cancer patients with COPD (COPD + LC group) who were admitted to the Department of Respiratory and Critical Care Medicine at the First Affiliated Hospital, Zhejiang University School of Medicine. The patients were selected consecutively from January 2021 to December 2022. All participants were aged 60 years or older. The follow-up date was until June 2023. Our study was conducted in compliance with ethical standards for research involving human subjects. The Ethics Board of the First Affiliated Hospital of Zhejiang University approved this study.

### Inclusion criteria

1. CPFE: Diagnosis was based on Cottin’s 2005 criteria [6], HRCT scans demonstrate emphysematous changes predominantly distributed in the upper lungs, characterized by the presence of low attenuation areas with thin walls (< 1 mm) and no clear boundaries, or multiple pulmonary bullae with diameters > 1 cm. The extent of pulmonary emphysema within the lung fields should be ≥ 10.0%. Additionally, HRCT of the chest should show fibrotic changes primarily involving the lower lungs and subpleural areas, characterized by a reticular pattern, as well as varying degrees of honeycombing and/or traction bronchiectasis.
2. COPD: Based on the GOLD 2023 guidelines [13], patients had a discharge diagnosis of COPD, with CT scans revealing increased and thickened pulmonary markings, bilateral lung fields with areas of low attenuation characterized by the absence of walls or extremely thin walls, or the presence of pulmonary bullae (diameter ≥ 1 cm, wall thickness ≤ 1 mm).
3. Lung cancer: Confirmation of tumor cells through biopsy, surgical pathology, or cytological examination, including lymph node biopsy, sputum cytology, or pleural fluid cytology. Patients exhibited an Eastern Cooperative Oncology Group (ECOG) performance status ranging from 0 to 2.

### Exclusion criteria

1. Excluding conditions such as nodular diseases, granulomatosis with polyangiitis, allergic alveolitis, lymphangioleiomyomatosis, eosinophilic pneumonia, Langerhans cell histiocytosis, pulmonary alveolar proteinosis, idiopathic pulmonary hemosiderosis, drug- or treatment-related interstitial changes (e.g., pesticides, radiation).
2. Excluding patients with lung metastases from other tumors, severe liver or kidney dysfunction, hematological malignancies, severe cardiovascular or cerebrovascular diseases, concurrent viral infections, asthma, or a large amount of pleural effusion/ pneumothorax.
3. Excluding patients with missing HRCT imaging and pulmonary function results during hospitalization, those whose lung cancer pathology cannot be classified, and those with incomplete medical records and relevant examination data.

### Data collection

The patient data was retrieved from the electronic medical records, including demographics, laboratory results, tumor pathology, TNM staging (the eighth edition) [14], tumor location, comorbidities, HRCT images, and pulmonary function test results. Document information of advanced cancer patients with TNM staging ranging from IIIB to IV. Take note of whether these patients underwent immunotherapy, record immunotherapy-related adverse reactions (AEs), document the initiation date of first-line and second-line chemotherapy, and make a record of the time of the first occurrence of disease progression (switch to second-line chemotherapy or imaging evidence of tumor progression) or death (whichever occurred first).

### Statistical analysis

Data analysis was conducted using SPSS version 25.0. Graphing was performed using GraphPad Prism 8. Categorical variables were summarized as frequencies and percentages, and continuous variables were described using mean ± standard deviation or median with interquartile range. We used the Mann-Whitney U test for continuous variables, and the Chi-squared(χ²) test or Fisher’s exact test for categorical data, with Bonferroni correction for multiple comparisons. The Log-rank test was used to compare survival differences between CPFE + LC group and COPD + LC group, and the Kaplan-Meier method was used to construct survival curves for progression-free survival (PFS). *P*<0.05 was considered statistically significant.

## Results

### Demographics and clinical comorbidities

As shown in Table 1, the CPFE + LC group consisted exclusively of male patients (100%, *n* = 75), while the COPD + LC group was predominantly male (99.45%, *n* = 182). The median age for both groups was 69 years. A significant disparity was observed in BMI, with the CPFE + LC group showing a higher average BMI (22.85 ± 3.43) compared to the COPD + LC group (21.51 ± 2.94), *p* = 0.002.

**Table 1:** Clinical characteristics and comorbidities in CPFE + LC and COPD + LC patients

Variable	CPFE + LC (*n* = 75)	COPD + LC (*n* = 182)	Statistic	*P*
Gender (men)	75 (100.00%)	181 (99.45%)	-	1.000
Age	69 (66,74)	69 (66,73)	Z=-0.413	0.679
BMI (kg/m²)	22.85 ± 3.43	21.51 ± 2.94	t=-3.147	0.002
ECOG (score)			χ²=3.828	0.148
0	46 (61.30%)	121 (66.50%)		
1	25 (33.30%)	59 (32.40%)		
2	4 (5.30%)	2 (1.10%)		
Ex- or current smokers	75 (100.00%)	176 (96.70%)	χ²=1.292	0.256
Smoking amount (pack-years)	40.00 (30.00–60.00)	40.00 (30.00–60.00)	Z = 0.428	0.671
Bronchiectasia	3 (4.00%)	18 (9.89%)	χ²=2.456	0.117
Pulmonary hypertension	12 (16.00%)	26 (14.29%)	χ²=0.124	0.725
Connective tissue disease	2 (2.67%)	4 (2.20%)	χ²=0.000	1.000
Coronary artery atherosclerosis	43 (57.33%)	89 (48.90%)	χ²=1.512	0.219
Hypertension	31 (41.33%)	71 (39.01%)	χ²=0.120	0.729
Diabetes mellitus	8 (10.67%)	23 (12.64%)	χ²=0.194	0.659
Previous pulmonary tuberculosis	1 (1.33%)	8 (4.40%)	χ²=0.707	0.400
Previous cerebral infarction	6 (8.00%)	1 (0.50%)	χ²=8.493	0.004
Pulmonary embolism	3 (4.00%)	4 (2.20%)	χ²=0.149	0.700
Peripheral vascular disease	6 (8.00%)	13 (7.10%)	χ²=0.057	0.811
Dust exposure	0 (0.00)	2 (1.10%)	-	1.000
Family history of cancer	7 (9.33%)	15 (8.24%)	χ²=0.081	0.776

BMI: body mass index; ECOG: Eastern Cooperative Oncology Group

The prevalence of smoking was high in both groups, with all the CPFE + LC patients and 96.70% of the COPD + LC patients being current or former smokers. No significant difference in smoking amount (pack-years) was observed. Comorbid conditions such as bronchiectasis, pulmonary arterial hypertension, connective tissue diseases, coronary artery atherosclerosis, hypertension, diabetes, prior pulmonary tuberculosis, pulmonary embolism and peripheral vascular disease had similar rates in both groups. However, the incidence of previous cerebral infarction was significantly higher in the CPFE + LC group, *p* = 0.004.

### Lung cancer pathology and radiological findings

There was a statistically significant difference in the pathology of lung cancer between the two groups (*p* = 0.004). Adenocarcinoma (36.00%) and squamous cell carcinoma (34.67%) were more common in CPFE, while squamous cell carcinoma (52.75%) was more common in COPD. The distribution of tumors differed between the two groups (*p* < 0.0167), with a higher incidence of lung cancer in the lower lobes (60.00%) in the CPFE + LC group compared to the COPD + LC group (40.66%). The CPFE + LC group also had a higher metastasis rate (49.33%) than the COPD + LC group (32.97%) (*p* = 0.014). It’s worth noting that nearly half of the patients in the CPFE + LC group were diagnosed with stage IV lung cancer (49.33%), whereas in the COPD + LC group, there were more patients at stage III (44.51%), though this difference was not statistically significant. Statistical analysis revealed that the types of emphysema were significantly different between the two groups (*p* < 0.001). Both panlobular emphysema and paraseptal emphysema exhibited intergroup differences in both groups (both *p* < 0.0167). Paraseptal emphysema was the most common type in the CPFE + LC group (41.33%), while centrilobular emphysema had the highest prevalence in the COPD + LC group (60.99%). The results are indicated in Table 2.

### Pulmonary function test parameters

As shown in Table 2, pulmonary function parameters such as FEV1 and FEV1/ FVC were higher in the CPFE + LC group (all *p* < 0.001). In contrast, DLCO (*p* < 0.001), DLCO/ VA (*p* = 0.009), RV (*p* < 0.001), and RV/ TLC (*p* < 0.001) were significantly lower in the CPFE+LC group compared to the COPD + LC group.

**Table 2:** Comparison of tumor characteristics, radiographic distribution, and pulmonary function between lung cancer patients with CPFE or COPD

Variable	CPFE + LC (*n* = 75)	COPD + LC (*n* = 182)	Statistic	*P*
Pathological classification			χ²=13.100	0.004
Adenocarcinoma	27 (36.00%)	64 (35.16%)		
Squamous cell carcinoma	26 (34.67%)	96 (52.75%)		
Small cell carcinoma	18 (24.00%)	19 (10.44%)		
Other	4 (5.33%)	3 (1.65%)		
Distribution of left/ right lung lobes			χ²=0.750	0.386
Right lung	44 (58.67%)	96 (52.75%)		
Left lung	31 (41.33%)	86 (47.25%)		
Distribution of lung lobe location			χ²=8.557	0.014
Upper lobe	26 (34.67%) ^*^	99 (54.40%)		
Middle lobe	4 (5.33%)	9 (4.95%)		
Lower lobe	45 (60.00%) ^*^	74 (40.66%)		
T			χ²=2.545	0.467
1	9 (12.00%)	28 (15.38%)		
2	24 (32.00%)	62 (34.07%)		
3	13 (17.33%)	19 (10.44%)		
4	29 (38.67%)	73 (40.11%)		
N			χ²=5.060	0.167
0	14 (18.67%)	34 (18.68%)		
1	5 (6.67%)	27 (14.84%)		
2	30 (40.00%)	77 (42.31%)		
3	26 (34.67%)	44 (24.18%)		
M			χ²=6.054	0.014
0	38 (50.67%)	122 (67.03%)		
1	37 (49.33%)	60 (32.97%)		
Stage			χ²=6.723	0.081
I	3 (4.00%)	16 (8.79%)		
II	8 (10.67%)	25 (13.74%)		
III	27 (36.00%)	81 (44.51%)		
IV	37 (49.33%)	60 (32.97%)		
Types of emphysema			χ²=30.028	< 0.001
Panlobular emphysema	24 (32.00%)	44 (24.18%)		
Centrilobular emphysema	20 (26.67%) ^#^	111 (60.99%)		
Paraseptal emphysema	31 (41.33%) ^#^	27 (14.84%)		
FEV1 (L)	1.92 ± 0.43	1.59 ± 0.54	t=-5.169	< 0.001
FVC (L)	2.69 ± 0.52	2.60 ± 0.64	t=-1.276	0.204
FEV1/ FVC (%)	71.19 (64.65–76.89)	61.25 (52.25–66.49)	Z = 7.206	< 0.001
VC (L)	2.30 (1.97–2.73)	2.21 (1.76–2.71)	Z = 1.105	0.269
DLCO (mL/min/mmHg)	8.48 (4.02–11.59)	10.11 (4.35–14.81)	Z = 2.398	0.016
DLCO/ VA (mL/min/mmHg/L)	2.02 (0.85–2.96)	2.69 (1.00–3.40)	Z = 2.595	0.009
RV (L)	1.97 (1.60–2.41)	2.35 (1.92–2.81)	Z = 3.945	< 0.001
TLC (L)	4.18 (3.70–4.84)	4.51 (3.74–5.20)	Z = 1.671	0.095
RV/ TLC (%)	47.20 (40.84–52.55)	52.11 (46.50–57.65)	Z = 4.605	< 0.001

^*^P < 0.0167 when compared to the COPD + LC group

^#^*P* < 0.0167 when compared to the COPD + LC group

T: tumor; N: node; M: metastasis; FEV1: forced expiratory volume in 1 s; FVC: forced vital capacity; FEV1/ FVC: forced expiratory volume in 1 s to forced vital capacity ratio; VC: vital capacity; DLCO: diffusing capacity of the lung for carbon monoxide; DLCO/ VA: diffusing capacity per unit of alveolar volume; RV: residual volume; TLC: total lung capacity; RV/ TLC: residual volume to total lung capacity ratio

### Laboratory test results

As shown in Table 3, the CPFE + LC group exhibited significantly higher levels of TG (*p* = 0.040), CEA (*p* = 0.010), NSE (*p* = 0.003), and killer T cell counts (CD3+, CD8+) (*p* = 0.028) compared to the COPD + LC group. But there were no statistically significant differences in complete blood count, immunoglobulins, complement, and inflammatory cytokines.

**Table 3:** Laboratory test results in lung cancer patients with CPFE or COPD

Variable	CPFE + LC (*n* = 75)	COPD + LC (*n* = 182)	Statistic	*P*
WBC (×10^9^/L)	6.60 (5.39–8.70)	6.63 (5.72–7.84)	Z = 0.641	0.521
PLT (×10^9^/L)	215.00 (172.50–275.00)	234.00 (188.25–296.00)	Z = 1.632	0.103
ANC (×10^9^/L)	4.40 (3.27–5.92)	4.38 (3.38–5.62)	Z = 0.256	0.798
ALC (×10^9^/L)	1.52 (1.19–1.86)	1.39 (1.02–1.78)	Z = 1.743	0.081
TG (mmoL/L)	1.16 (0.87–1.60)	0.99 (0.75–1.43)	Z = 2.053	0.040
TC (mmoL/L)	3.93 (3.30–4.76)	4.17 (3.54–4.74)	Z = 1.272	0.203
LDL (mmoL/L)	2.07 (1.77–2.83)	2.31 (1.84–2.83)	Z = 1.103	0.270
CRP (mg/L)	8.60 (3.20–20.67)	4.77 (2.45–23.50)	Z = 1.319	0.187
Fibrinogen (g/L)	3.67 (2.87–4.93)	3.58 (2.96–4.78)	Z = 0.232	0.817
C4 (mg/dL)	27.66 ± 7.95	29.47 ± 9.25	t = 0.774	0.441
C3 (mg/dL)	121.32 ± 28.13	119.28 ± 22.97	t=-0.323	0.747
IgG (mg/dL)	1302.32 ± 395.23	1201.21 ± 311.91	t=-1.171	0.245
IgM (mg/dL)	79.00 (51.00–97.00)	92.10 (59.25–127.25)	Z = 1.276	0.202
IgA (mg/dL)	281.00 (190.00–310.00)	195.50 (156.75–283.50)	Z = 1.561	0.118
CEA (ng/mL)	6.20 (3.50–9.95)	4.30 (3.00–7.97)	Z = 2.591	0.010
Cyfra 21 − 1 (ng/mL)	3.80 (2.20–7.62)	3.00 (1.90–6.70)	Z = 1.839	0.066
NSE (ng/mL)	18.40 (13.35–26.48)	15.60 (12.83–20.00)	Z = 2.997	0.003
SCC (ng/mL)	1.20 (1.00–2.27)	1.20 (0.80–2.00)	Z = 0.585	0.559
IL-2 (pg/mL)	0.70 (0.31–1.47)	1.02 (0.34–1.96)	Z = 1.442	0.151
IL-4 (pg/mL)	1.12 (0.27–1.83)	1.23 (0.36–2.34)	Z = 1.490	0.137
IL-6 (pg/mL)	10.27 (5.76–24.79)	7.98 (4.33–22.88)	Z = 0.880	0.379
IL-10 (pg/mL)	1.87 (1.03–3.20)	2.00 (1.43–3.97)	Z = 1.202	0.229
TNF-α (pg/mL)	2.04 (0.99–3.72)	2.49 (1.17–4.89)	Z = 1.443	0.149
IFN-γ (pg/mL)	1.67 (0.36–5.22)	2.72 (0.89–5.03)	Z = 1.441	0.149
IL-17 A (pg/mL)	1.15 (0.10–9.66)	2.23 (0.10–12.06)	Z = 0.926	0.373
T cells (CD3+) (%)	67.27 ± 12.58	67.82 ± 10.40	t = 0.283	0.778
T cells count (CD3+)(cell/µL)	917.50 (715.50–1105.75)	854.00 (571.75–1122.50)	Z = 1.350	0.177
Helper T cells (CD3+,CD4+) (%)	36.41 ± 11.82	39.16 ± 10.53	t = 1.420	0.158
Helper T cells count (CD3+,CD4+)(cell/µL)	520.00 (348.00–665.50)	477.50 (320.50–631.25)	Z = 0.880	0.379
B cells (CD19+) (%)	8.78 (5.09–13.40)	8.48 (5.50–12.22)	Z = 0.130	0.897
B cells count (CD19+)(cell/µL)	110.00 (64.00–192.75)	96.00 (60.75–148.50)	Z = 0.884	0.377
Killer T cells (CD3+,CD8+) (%)	28.62 (17.56–34.87)	24.10 (18.24–32.07)	Z = 0.971	0.331
Killer T cells count (CD3+,CD8+) (cell/µL)	367.50 (230.50–490.75)	281.50 (186.75–415.75)	Z = 2.193	0.028
NK cells (CD16+, CD56+) (%)	19.45 (12.85–30.20)	20.00 (13.98–29.88)	Z = 0.353	0.724
NK cells count (CD16+, CD56+) (cell/µL)	256.50 (153.50–413.25)	241.50 (147.50–343.50)	Z = 0.917	0.359
Lymphocyte count (CD45+)(cell/µL)	1382.50 (1189.25–1731.25)	1232.00 (894.75–1635.00)	Z = 1.867	0.062
CD4+/ CD8 + ratio	1.44 (0.95–2.14)	1.65 (1.03–2.34)	Z = 1.135	0.256

WBC: white blood cells; PLT: platelets; ANC: absolute neutrophil count; ALC: absolute lymphocyte count; TG: triglycerides; TC: total cholesterol; LDL: low-density lipoprotein; CRP: C-reactive protein; C4: complement 4; C3: complement 3; IgG: immunoglobulin G; IgM: immunoglobulin M; IgA: immunoglobulin A; CEA: carcinoembryonic antigen; Cyfra 21 − 1: cytokeratin 19 fragment; NSE: neuron-specific enolase; SCC: squamous cell carcinoma antigen; IL-2: interleukin 2; IL-4: interleukin 4; IL-6: interleukin 6; IL-10: interleukin 10; TNF-α: tumor necrosis factor alpha; IFN-γ: interferon gamma; IL-17 A: interleukin 17 A

**Fig. 1:**
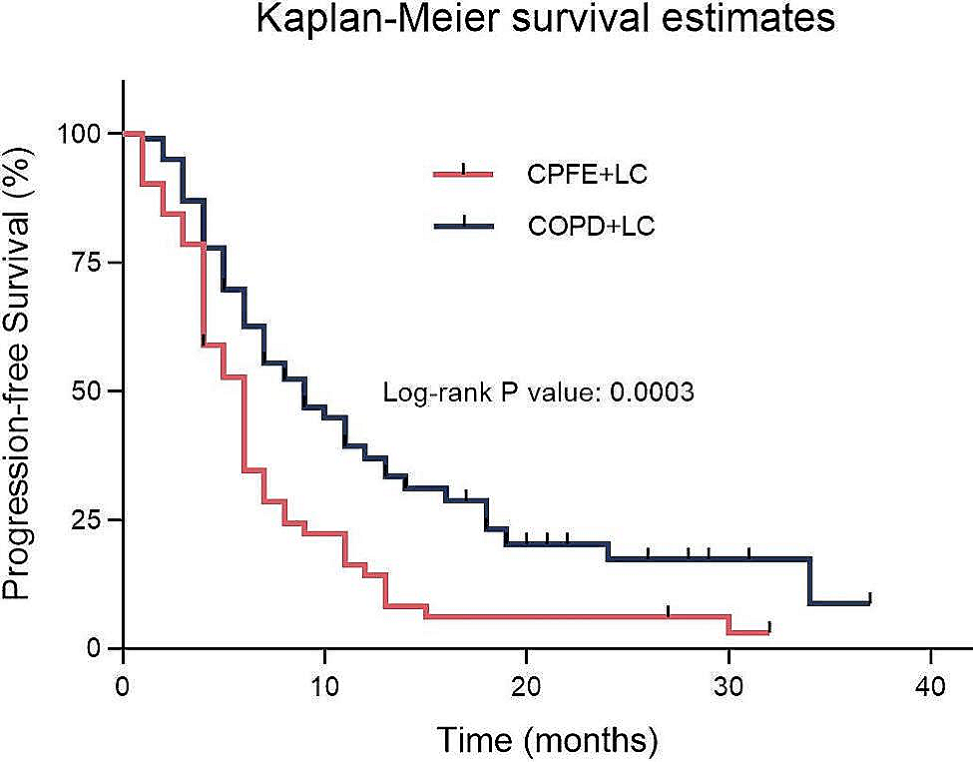
Comparison of PFS between lung cancer patients with CPFE or COPD

We have compiled the PFS data of the first-line treatment for all advanced cancer patients with TNM staging ranging from IIIB to IV. The results indicate that in the CPFE + LC group, comprising 51 patients, the median PFS of first-line treatment was 6.0 months (95% CI: 4.9–7.1). In contrast, the COPD + LC group, consisting of 99 patients, exhibited a longer median PFS of 9.0 months (95% CI: 6.2–11.8). Statistical analysis revealed a significant difference in PFS between the two groups (*p* = 0.0003, χ²=13.29). Comparing the CPFE + LC group to the COPD + LC group, the former showed an 85.7% increased risk of disease progression or death after receiving first-line chemotherapy, with a hazard ratio (HR) of  1.857 (95% CI: 1.239–2.783). Figure 1 illustrates Kaplan- Meier survival curves for two groups undergoing pharmacological treatment.

**Fig. 2:**
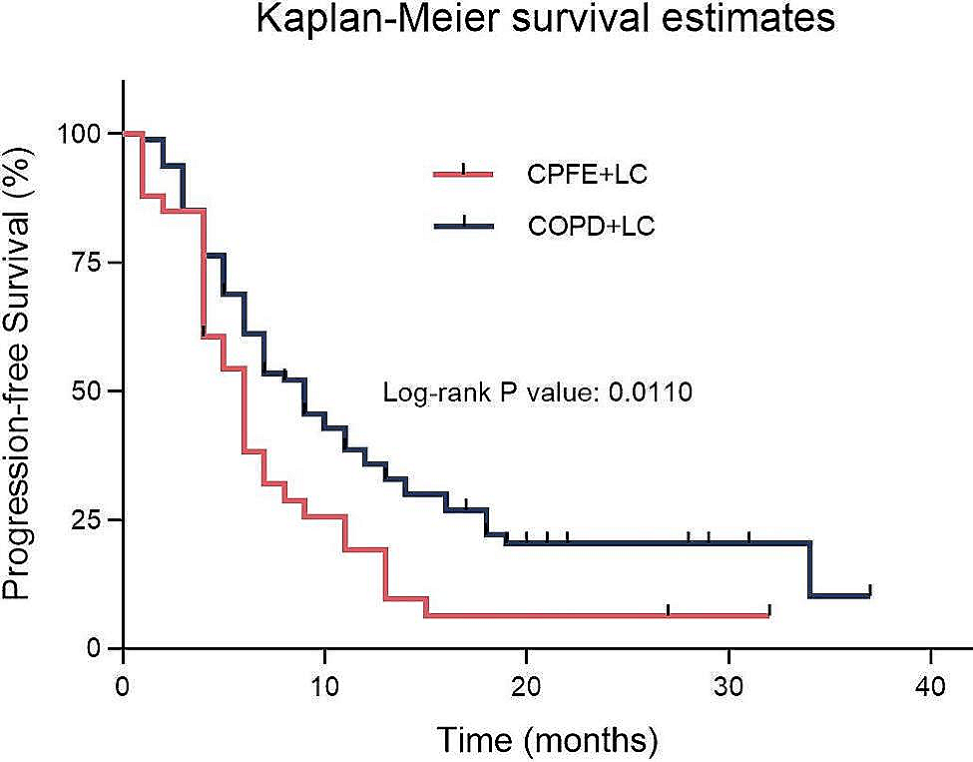
Comparison of PFS between NSCLC patients with CPFE or COPD

In all advanced lung cancer patients with TNM staging ranging from IIIB to IV who received first-line treatment, we conducted subgroup survival analyses separately for patients with non-small cell lung cancer (NSCLC) and small cell lung cancer (SCLC). In the CPFE + LC group, consisting of 33 NSCLC patients, the median PFS was 6.0 months (95% CI: 4.491–7.509). In the COPD + LC group, comprising 80 NSCLC patients, the median PFS was 9.0 months (95% CI: 6.597–11.403). Statistical analysis revealed a significant difference in PFS between the two groups (*p* = 0.0110, χ²=6.464). After receiving first-line treatment, the CPFE + LC group showed a 69.3% increased risk of disease progression or death, with a HR of 1.693 (95% CI: 1.035–2.770). Figure 2 displays the Kaplan- Meier survival curves for the two groups of NSCLC patients following pharmacological treatment.

**Fig. 3:**
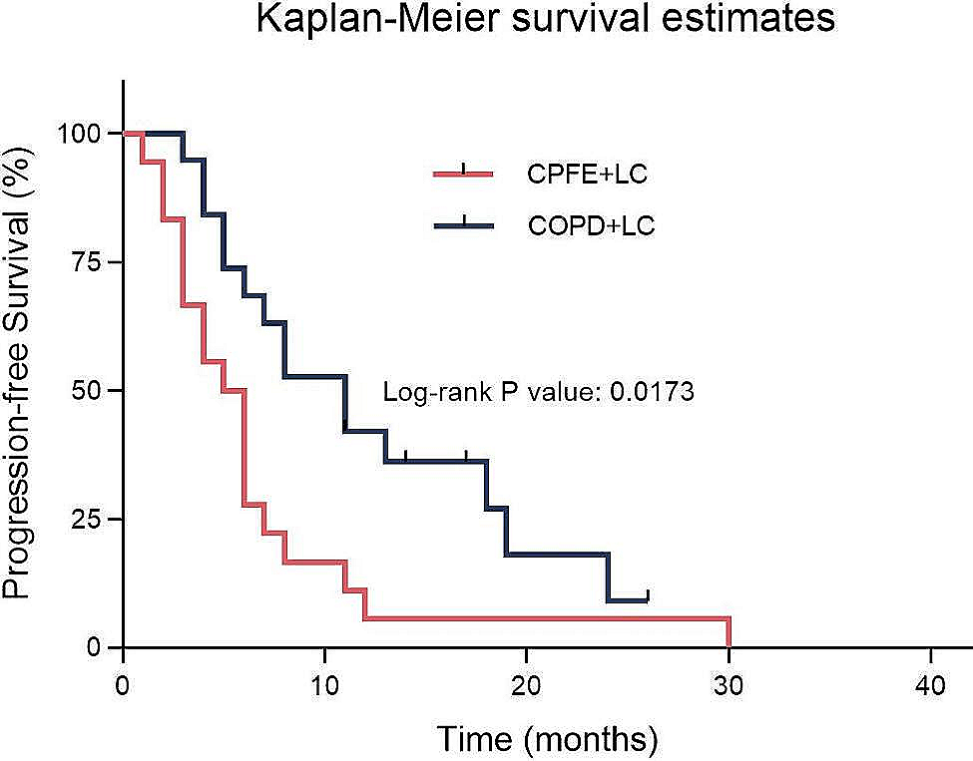
Comparison of PFS between SCLC patients with CPFE or COPD

In the CPFE + LC group, consisting of 18 SCLC patients, the median PFS was 5.5 months (95% CI: 3.337–6.663). In the COPD + LC group, comprising 19 SCLC patients, the median PFS was 11.0 months (95% CI: 6.782–15.218). Statistical analysis indicated a significant difference in PFS between the two groups (*p* = 0.0173, χ²=5.667). Following first-line treatment, the CPFE + LC group exhibited a 109.1% increased risk of disease progression or death, with a HR of 2.091 (95% CI: 1.029–4.247). Figure 3 displays the Kaplan- Meier survival curves for the two groups of SCLC patients receiving pharmacological treatment.

**Fig. 4:**
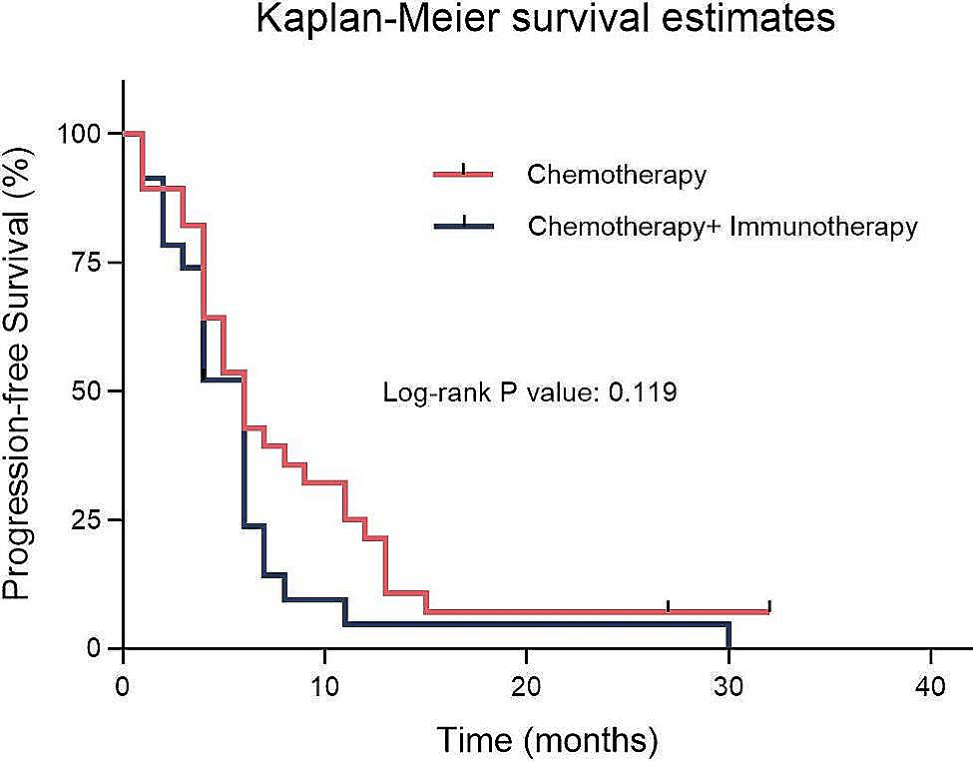
Comparative analysis of PFS with different treatment modalities in CPFE + LC patients

We further analyzed the 51 patients in the CPFE + LC group mentioned above. Among them, 23 patients received chemotherapy as first-line treatment, with a median PFS of 6.0 months (95% CI: 4.9–7.1). The remaining 28 patients received a combined treatment with immunotherapy and chemotherapy, and their median PFS of first-line treatment was 6.0 months (95% CI: 4.3–7.7), with an HR of 0.672 (95% CI: 0.373–1.211) (*p* = 0.119, χ²=2.42). These results indicate that, in the CPFE + LC group, there was no significant difference in the median PFS of first-line treatment between the chemotherapy-only group and the combined treatment group. Figure 4 illustrates the Kaplan- Meier survival curves for two treatment groups within the CPFE + LC group.

Among the CPFE + LC group, consisting of 28 patients who received immunotherapy, 10 cases (35.7%) reported immune-related AEs. In contrast, among the 78 lung cancer patients with COPD who were treated with immunotherapy, 19 cases (24.4%) experienced immune-related AEs. Statistical analysis indicated no significant difference between the two groups in terms of AEs (*p* = 0.248, χ²=1.337).

The CPFE + LC group exhibited a higher incidence of interstitial pneumonia (50.0%) and pituitary insufficiency (20.0%). Conversely, the COPD + LC group predominantly experienced interstitial pneumonia (36.8%) and liver dysfunction (15.8%). The incidence and specific immune-related adverse events in the two groups are presented in Table 4.

**Table 4:** Comparison of incidence and specific immune-related adverse events in CPFE + LC versus COPD + LC patients

	CPFE + LC (*n* = 28)	COPD + LC (*n* = 78)	Statistic	*P*
Immune-related Adverse Events	10 (35.7%)	19 (24.4%)	χ²=1.337	0.248
Interstitial pneumonia	5 (50.0%)	7 (36.8%)		
Pituitary insufficiency	2 (20.0%)	0		
Liver dysfunction	0	3 (15.8%)		
Renal insufficiency	1 (10.0%)	2 (10.5%)		
Diarrhea	0	2 (10.5%)		
Rash	1 (10.0%)	1 (5.3%)		
Cystitis	1 (10.0%)	0		
Myositis	0	1 (5.3%)		
Intracranial edema	0	1 (5.3%)		
Pancreatitis	0	1 (5.3%)		
Hearing loss	0	1 (5.3%)		

## Discussion

This retrospective study reveals the following findings: The CPFE + LC group has higher levels of BMI, TG, CEA, NSE, and Killer T cells count compared to the COPD + LC group. Lung cancer in the CPFE group primarily manifests as adenocarcinoma and squamous cell carcinoma, predominantly located in the lower lung lobes, with a higher rate of tumor metastasis and a main characteristic of paraseptal emphysema. In contrast, lung cancer in the COPD group is mainly squamous cell carcinoma, mainly found in the upper lung lobes, and associated with centrilobular emphysema. In advanced cancer patients with TNM staging ranging from IIIB to IV, the CPFE + LC group has a shorter median PFS than the COPD + LC group after first-line treatment. Patients with either NSCLC or SCLC who have combined CPFE have a worse prognosis than those with combined COPD. Within the CPFE + LC group, patients may not derive additional benefits from immunotherapy. And there is no significant difference in the incidence of immune-related AEs between the CPFE + LC group and the COPD + LC group receiving immunotherapy.

After conducting a comparative analysis of CPFE + LC and COPD + LC patients, we noticed that the primary common characteristics in both groups were elderly male patients, with the majority having a history of smoking. Additionally, we observed relatively higher BMI and TG levels in the CPFE + LC group. This finding sparked our interest as it may be related to the presence of pulmonary fibrosis. Seeliger et al.‘s study [15] also mentioned significantly elevated triglyceride levels in patients associated with interstitial lung diseases, such as pulmonary fibrosis. This provides some support for our observations. Furthermore, the treatment of pulmonary fibrosis may involve the use of glucocorticoids, which could also be linked to the higher BMI and TG levels.

Smoking can impact lung function and affect the clinical symptoms of patients with CPFE and COPD [16, 17]. During our investigation into the differences in lung function between CPFE + LC and COPD + LC patients, we observed that CPFE + LC group had lower values for indicators such as DLCO, DLCO/ VA, RV, and RV/ TLC compared to the COPD + LC group. This indicated a predominant impairment in gas exchange, which is typically associated with pulmonary fibrosis and emphysema. Conversely, the COPD + LC group exhibited worse results in parameters such as FEV1 and FEV1/ FVC. These lung function results reflect the characteristics of the two primary diseases, suggesting that the presence of lung cancer does not lead to significant changes in lung function.

The increased susceptibility of lung cancer in CPFE or COPD patients may primarily be attributed to a combination of factors such as chronic inflammation [18–20], DNA damage [21], and impaired apoptosis function [22, 23]. Additionally, genetic predisposition and occupational exposures [24] can also influence the development of lung cancer. Our research findings suggested that the majority of lung cancer patients with CPFE or COPD have a history of smoking. Smoking-induced oxidative stress can lead to lipid peroxidation and DNA damage [25]. It can also induce global changes in gene methylation status and potentially impact genes involved in cell cycle regulation, airway remodeling, wound healing, and more [26, 27], thus promoting carcinogenesis and increasing the risk of lung cancer in patients with a smoking history.

Our study indicated that the proportions of adenocarcinoma (36.00%) and squamous cell carcinoma (34.67%) were quite similar among CPFE patients, while in COPD patients, squamous cell carcinoma was the predominant lung cancer type, accounting for 52.75% of cases. Consistent with our findings, a study by Usui et al. [28] in Japan on CPFE patients with concurrent lung cancer also found a higher proportion of adenocarcinoma (45.5%) compared to squamous cell carcinoma (30.7%). However, in an extensive pathological examination of 47 CPFE patients with concurrent lung cancer, Girard et al. found that 38 of them (81%) had lung cancer, with 17 cases (36%) being squamous cell carcinoma and 14 cases (30%) being adenocarcinoma [29]. Additionally, Koo et al. [30] conducted a meta-analysis and reported that CPFE combined with lung cancer primarily occurs in elderly males with a history of smoking, with squamous cell carcinoma being the predominant type. The viewpoint that COPD patients are more prone to squamous cell carcinoma is supported by numerous studies. For instance, Bozinovski et al.’s analysis [31] suggests that abnormal inflammation and immune responses are common underlying factors in COPD patients’ susceptibility to squamous cell carcinoma. Zhang et al. [32] confirmed through a Mendelian randomization study that airflow limitation (FEV1/ FVC < 0.7) is an independent predictor for lung squamous cell carcinoma. Liu et al. [33] conducted an analysis of patients with IPF and concurrent lung cancer, revealing that 45.65% of IPF patients had adenocarcinoma.

Our research revealed that CPFE patients were more prone to develop lung cancer in the lower lobes of the lungs, particularly in areas affected by pulmonary fibrosis. This pattern accounted for 60% of cases in the CPFE + LC group. In contrast, COPD patients tended to have lung cancer occurrences predominantly in the upper lobes of the lungs. The differences in the locations of lung cancer in these two groups were statistically significant. Kwak et al.’s study [34] also supports the notion that lung cancer associated with CPFE is more likely to occur in the subpleural region, closer to dense fibrotic areas. This finding aligns with the conclusion reached by Liu et al. [33], who found that lung cancer in IPF patients primarily occurs in the peripheral and lower lobes, consistent with the affected areas of IPF. Additionally, Bae et al. [35] reported that in COPD patients, lung cancer is most likely to occur in the upper lobes of both lungs, with an odds ratio of 1.77 when compared to the lower/ middle lobes. This observation implies that emphysema may not have an additional impact on CPFE- related lung cancer and lends support to the potential relationship between cancer development and fibrotic regions. In addition to these factors, the subtypes of emphysema are also correlated with lung cancer [36].

The imaging findings of CPFE typically include upper-lobe emphysema and lower-lobe interstitial fibrotic pattern. Previous studies have shown differences in the distribution of the emphysema types between CPFE and COPD. The emphysematous change in COPD is usually centrilobular, while in CPFE paraseptal emphysema is much more frequent [5, 37–39]. In our study, among CPFE + LC patients, paraseptal emphysema was the most common subtype, accounting for 41.33% of cases, while in COPD + LC patients, centrilobular emphysema was more prevalent, making up 60.99% of cases, which was in accordance with previous studies. Oikonomou et al’s study [40] demonstrated that CPFE patients with paraseptal emphysema most commonly show a higher extent of fibrosis with a UIP pattern, while centrilobular emphysema may be associated with a higher extent of emphysema and an NSIP pattern, indicating a stronger association of paraseptal emphysemas with typical UIP pattern of fibrotic change. Moreover, it is worth noting that some research has found that the presence of paraseptal emphysema increases the risk of adenocarcinoma in COPD patients [41]. However, González et al. [42] discovered in a lung cancer screening project in Spain that airflow obstruction is associated with an increased risk of lung cancer, but this risk is reduced in the presence of paraseptal emphysema. This may help explain why paraseptal emphysema is less prevalent in COPD + LC patients.

For patients in the CPFE + LC group, regardless of whether they received chemotherapy alone or a combination of chemotherapy and immunotherapy, the median PFS of first-line treatment was 6.0 months. Our research reveals that lung cancer patients with CPFE may not derive additional benefits from immunotherapy. Tan et al. [43] reported two cases of lung cancer patients with CPFE, both of whom received a treatment of chemotherapy combined with immunotherapy. After treatment, both patients experienced a significant reduction in tumor size. However, one eventually died from worsening acute interstitial lung disease caused by immunotherapy, while the other died due to tumor infiltration after discontinuing immunotherapy. Our study found that 35.7% of the CPFE + LC group receiving immunotherapy experienced immune-related AEs, while the COPD + LC group accounted for 24.4%. There was no significant difference in the incidence of immune-related AEs in two groups, indicating similar tolerability to immunotherapy in both groups. We also found that both the CPFE + LC group and the COPD + LC group had interstitial pneumonia as the predominant immune-related AE. Immune checkpoint inhibitors can lead to immune-related AEs, with interstitial lung disease being one of the more severe adverse events among them [44].

In our study, we observed that the CPFE + LC group exhibited significantly higher levels of tumor markers such as CEA and NSE compared to those in the COPD + LC group. These elevated levels of tumor markers may suggest a greater tumor burden or more extensive tumor invasion. Furthermore, in accordance with the research by Koo et al. [30], lung cancer in CPFE patients is often diagnosed at advanced stages, possibly due to fibrosis and emphysema masking the symptoms of the tumor, resulting in misdiagnosis or delayed diagnosis. This not only exacerbates the already compromised lung function but also increases the complexity of surgical treatment. In addition, numerous studies have identified common signaling pathways in lung cancer and pulmonary fibrosis. For example, connexin 43 has been found to exhibit reduced expression or expression loss in both conditions [45]. Additionally, molecules involved in the regulation of the Wnt/ beta-catenin signaling pathway are overexpressed in the lung tissues of patients with lung cancer and pulmonary fibrosis, contributing to processes such as lung remodeling and carcinogenesis [46].

We found that in advanced cancer patients with TNM staging ranging from IIIB to IV, the CPFE + LC group receiving first-line treatment had a significantly shorter median PFS of 6.0 months compared to 9.0 months in the COPD + LC group. This trend of poorer prognosis was consistently observed across different histological types: patients with NSCLC who had combined CPFE exhibited worse outcomes than those with combined COPD, and a similar prognostic pattern was observed in patients with SCLC. These results further emphasize the greater challenges faced by the CPFE + LC group in terms of treatment response and disease progression. Existing research indicates that CPFE + LC patients have a significantly shorter median survival compared to patients with lung cancer alone or lung cancer with emphysema [28]. Kumagai et al. [47] further revealed that CPFE patients exhibit lower tolerance to tumor chemotherapy, with a higher recurrence rate and markedly shorter Overall Survival (OS) in NSCLC.

Our study has several limitations. First, as a single-center, retrospective analysis, the generalizability of our findings may be limited and subject to selection bias. Second, we excluded patients who underwent surgical treatments, some of whom received neoadjuvant or adjuvant chemotherapy due to their earlier TNM stages, potentially skewing treatment efficacy results. Furthermore, the brief study period and small sample size may have introduced bias. For example, the fact that all patients in the CPFE + LC group were male could indicate a selection bias. Also, the lower incidence of CPFE compared to COPD resulted in a small number of lung cancer patients with CPFE, leading to a numerical imbalance that might bias the results. Future studies should expand the sample size and include detailed survival analyses for each histological type of lung cancer. Finally, the short follow-up duration limited our ability to assess overall survival, restricting our analysis to PFS within the available follow-up period.

## Conclusion

In conclusion, this retrospective study underscores the distinct clinical, pathological, and functional characteristics of lung cancer patients with CPFE or COPD. Notable differences were observed in tumor pathology, pulmonary function parameters, and treatment responses between the CPFE + LC and COPD + LC groups. These findings highlight the necessity for tailored diagnostic and therapeutic approaches in managing lung cancer within these patient populations. While our study provides valuable insights, its retrospective, single-center nature suggests the need for further multicenter, prospective research to validate and expand upon these findings. Ultimately, this study emphasizes the critical need for heightened clinical awareness and individualized treatment strategies for lung cancer in the context of CPFE or COPD.

## Data Availability

The datasets generated and/or analyzed during the current study are not publicly available but are available from the corresponding author on reasonable request.

## Abbreviations

LC: Lung cancer
CPFE: Combined pulmonary fibrosis and emphysema
COPD: Chronic obstructive pulmonary disease
PFS: Progression-free survival
NSCLC: Non-small cell lung cancer
SCLC: Small cell lung cancer
IPF: Idiopathic pulmonary fibrosis
HRCT: High-resolution computed tomography
ECOG: Eastern Cooperative Oncology Group
AEs: Adverse reactions
HR: Hazard ratio
OS: Overall survival
